# Occupancy dynamics of free ranging American mink (*Neogale vison*) in Greece

**DOI:** 10.1038/s41598-024-60542-4

**Published:** 2024-04-30

**Authors:** Dimitrios E. Bakaloudis, Charalambos T. Thoma, Konstantina N. Makridou, Evangelos G. Kotsonas

**Affiliations:** School of Forestry and Natural Environment, Aristotle University of Thessaloniki, P.O. Box 241, 541 24 Thessaloníki, Greece

**Keywords:** Invasive species, Invasive species

## Abstract

Identifying the environmental factors that determine the occurrence of invasive species is essential in defining and implementing effective control campaigns. Here, we applied multi-season occupancy models to analyze American mink (*Neogale vison*) track data collected using 121 floating rafts, as a function of factors occurring at multiple spatial scales. Our overall aim was to identify those factors that determine the use, colonization or abandonment of rafts by free ranging individuals found in western Macedonia, Greece. We found that increasing values of shrubs and rock cover at the micro-habitat scale were positively associated with the species’ probability of raft use, as was the density of medium-sized rivers at the landscape scale. Colonization was found to increase with increasing amounts of shrub and reed cover; however, both variables were not informative. Conversely, the distance from the nearest fur farm was highly informative in predicting raft abandonment by the species. Effective control actions may require removal by trapping along rocky or densely vegetated riverbanks or lake shores located in the vicinity of the established fur farms in the area. Habitat management, although possible, may be difficult to implement due to the ability of the species to adapt. Finally, fur farms should maximize security and establish an early warning and rapid eradication system in case of future escapes.

## Introduction

Over the past two centuries, human activities have led to a significant surge in the translocation of species outside their native range^1–3^. While many of these species play a crucial role in our global economy, the negative impacts of nonnative species invasions on native biodiversity are profound^4,5^. In Europe, the rate of invasion by nonnative species is expected to increase in the near future^5^. As a consequence, comprehending the mechanisms and patterns of biotic invasions has been the focus of many recent studies.

Native to North America, the American mink (*Neogale vison*) is a generalist, territorial, semiaquatic carnivore^6^. The species was first introduced to Europe in the 1920s as a furbearer^7^ and has since colonized many parts of the world through accidental and deliberate releases^8^. Among European countries, Greece holds one of the largest fur industries, with mink being bred for their fur since the 1970s^9^. Currently, there are more than 100 mink fur farms operating in the general region of western Macedonia^10^.

Feral mink populations are believed to have been established in western Macedonia following deliberate releases by so-called animal rights activists. More specifically, in 2010, approximately 50,000 mink were released, followed by another 10,000 in the year ahead. While most individuals were either recaptured or killed by passing vehicles, an unknown number of individuals became naturalized and were able to spread to the surrounding landscape through the Aliakmonas River valley. Despite the immense amount of scientific evidence suggesting that the American mink may cause conservation problems for local species^11–15^, no measures were taken at that time to prevent further spread or protection of native fauna. For example, mink may decimate entire colonies and populations of ground nesting birds, including several species of the *Anatidae* and *Rallidae* family^16^, many of which can be found in the general region of western Macedonia. Other affected species include reptiles and amphibians commonly found in riparian ecosystems^7,17^, whereas it may also affect directly or indirectly other aquatic predators such as the river otter (*Lutra lutra*)^18,19^, which is also present in the wider region. However, it was not until 2019 that a LIFE project (LIFE18 NAT/GR/000430) addressed this issue, following the example of many other European countries that have control policies and eradication campaigns focused on American mink^7^.

Although the ecology of mink outside its native range is well documented, information is lacking in southeastern Europe^20^. Previous studies have shown that mink can occupy areas as far as ~ 1 km away from water sources^21^, moving along banks with dense vegetation and complex ground structures where they can hide or den in, while they avoid open areas and high slopes^22–27^. In addition, mink presence may be influenced by biotic factors such as prey availability, competition and predation^28–30^. However, due to its ability to adapt to novel environments, the factors associated with its presence may vary among different regions. In Greece, the species has been poorly studied. In a recent study, Galanaki and Kominos^31^ investigated mink distribution. However, their findings were based on opportunistic data and offered little to no information regarding the factors driving mink presence in the region. In addition, Vada et al*.*^20^ concluded that in order to align management objectives and coordinate mink control across countries, mink monitoring should follow a standardized protocol at the continental scale.

In this study, we applied multi-season occupancy models to analyze American mink detection/non-detection data collected using 121 floating rafts, as a function of factors occurring at multiple spatial scales. Our overall aim was to identify those factors that determine the use, colonization or abandonment of rafts by free ranging individuals found in western Macedonia, Greece, and provide management recommendations for controlling the species.

## Methods

### Study area

**Figure 1:**
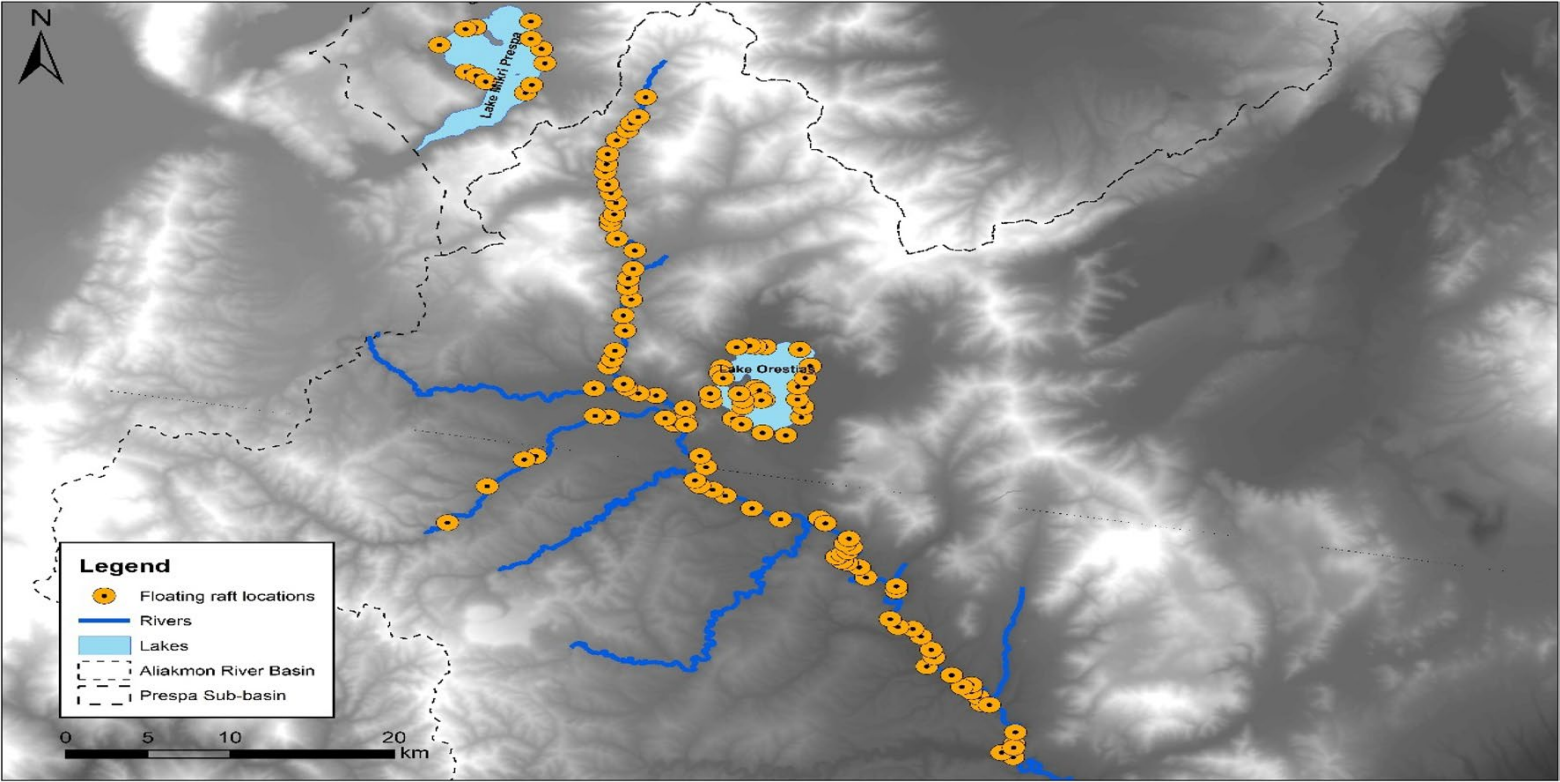
Study area and site locations of the 121 floating rafts used to track American mink presence in western Macedonia, Greece, 2021–2023.

The study was carried out between 2021 and 2023 within the Aliakmon River Basin and the Prespa Sub-basin. The Aliakmon River Basin included approximately 140 km stretches of river as well as lake Orestias. whereas the Prespa Sub-basin only included the Lake Mikri Prespa (Fig. 1). The Aliakmon River Basin covers an area of approximately 11,000 km^2^ and contains Greece’s largest river, the Aliakmon River^32^. The area is principally covered by forests and seminatural areas (57%), followed by agricultural areas (37.6%), urban areas (3.3%), water and wetlands (2.1%). Similarly, the Prespa Sub-basin covers an area of 347 km^2^ and is principally covered by forests (62%), followed by water (23.5%), agricultural land (7.7%), pastures (1.4%), and urban areas (1.1%)^33^.

### Floating raft surveys

American mink detection/non-detection data were gathered from track surveys carried out with the use of mink floating rafts, following pre-established protocols^34^, and in accordance with Greece animal welfare legislation. A detailed description of the floating raft can be found in Reynolds et al.^34^. In brief, a floating raft acts as a monitoring device by recording footprints on a clay cartridge. A total of 150 rafts were established within the study area, however data from 121 rafts were included in the analysis. Rafts were spaced at approximately 1 km intervals (mean = 823 m; range: 183–3965 m) along the Aliakmon River and its tributaries, as well as around Lakes Orestias and Mikri Prespa. Spacing between rafts was based on three different criteria; increase the probability of an individual being captured^35,36^, previous knowledge of mink home range size in Mediterranean regions^37^, and ease of access by the observers. Rafts were left in place for the entire study period. All tracks from each visit were photographed by the observers and sent to experts for identification. Data were recorded as mink presence or absence, depending on whether we found an imprint of mink paws on each clay cartridge during each visit.

Raft surveys were conducted in four distinct primary periods: September–November 2021, April–May 2022, October–December 2022, and March–April 2023. Each primary period was concluded within a time window of 25 days, during which rafts were set to record mink tracks for five consecutive days, followed by a 5-day trapping campaign at selected rafts (with recorded mink presence and immediate adjacent ones), and so on and so forth. Hence, during each primary period we recorded mink presence or absence in three distinct secondary periods, each of which lasted five consecutive days (total of 15 days of tracking required for each primary period).

### Ethical statement

All procedures carried out in this study were conducted in accordance with Greece animal welfare legislation, approved by the General Secretariat of Natural Environment and Water, Ministry of Environment and Energy, Hellenic Republic (Licence number: 75935/1924/16-12-2020) and conducted under the authority of the project licence (LIFE18 NAT/GR/000430).

### Environmental covariates

To better understand the associations between habitat features and mink occupancy, we followed a multiscale approach that included micro-habitat, local and landscape scale variables. At the micro-habitat scale, we used a 10 m transect along each of the main cardinal directions to estimate vegetation cover and cover of different ground components at each raft location^21,26^. Vegetation cover was estimated as the percent cover of three different strata (Table 1), with data recorded every 2 m. The percent cover of different ground components included bare soil, rocks, grass, shrubs and reeds, with data recorded every 50 cm (Table 1). Finally, we extracted elevation values at each site using a hand-held GPS device. Local and landscape variables were created using ArcGIS Desktop: Release 10^38^ and cartographic data from the Copernicus Land Monitoring Services. Variables included the percent cover of different land uses, river density and distance to the nearest fur farms (Table 1). Local variables referred to a distance class of 250 m around each raft, whereas landscape variables referred to a distance class of 1 km around each raft. All of the above variables were also used to account for temporal variation in colonization and extinction, along with some yearly covariates which included the effect of primary period (S), whether trapping was attempted or not (trap), and the number of individuals caught (catch) (Table 1). Finally, to account for imperfect detection, we included four observation-level covariates (Table 1). Prior to model fitting, all variables were checked for collinearity and were standardized to a mean of zero and a variance of one. Because variables “Strata 1” and percent (%) shrub cover (SH) were strongly correlated (Pearson’s r > 0.6), variable “Strata 1” was excluded from further analyses.

**Table 1:** Variables used in multi-season occupancy models for the American mink in western Macedonia, Greece, 2021–2023.

Parameter	Variable	Description	Scale/class
Initial probability of raft use	DEM	Elevation	Micro-habitat
	Strata1	Percent (%) vegetation cover (< 1 m height)	Micro-habitat
	Strata2	Percent (%) vegetation cover (1–5 m height)	Micro-habitat
	Strata3	Percent (%) vegetation cover (> 5 m height)	Micro-habitat
	BS	Percent (%) cover of bare soil	Micro-habitat
	R	Percent (%) cover of rocks/boulders	Micro-habitat
	GS	Percent (%) cover of grass	Micro-habitat
	SH	Percent (%) cover of shrubs	Micro-habitat
	RE	Percent (%) cover of reeds	Micro-habitat
	Arable	Percent (%) cover of arable land (includes levels 211, 212 and 213 of Corine Land Cover)	Local (250 m) and Landscape (1 km)
	HetAgri	Percent (%) cover of heterogenous agricultural land (includes levels 211, 243 and 244 of Corine Land Cover)	Local (250 m) and Landscape (1 km)
	Shrub	Percent (%) cover of shrubs (includes levels 321, 322, 323 and 324 of Corine Land Cover)	Local (250 m) and Landscape (1 km)
	Broad	Percent (%) cover of broadleaved forests (includes level 311 of Corine Land Cover)	Local (250 m) and Landscape (1 km)
	Dev	Percent (%) cover of developed areas (extracted from the layer “Build-up areas” of Copernicus)	Local (250 m) and Landscape (1 km)
	Riv	Density (km/km^2^) of all river classes (extracted from the layer “EU Hydro” of Copernicus)	Local (250 m) and Landscape (1 km)
	SRiv	Density (km/km^2^) of small rivers (Stahler 1 and 2) (extracted from the layer “EU Hydro” of Copernicus)	Landscape (1 km)
	Mriv	Density (km/km^2^) of medium rivers (Stahler 3, 4 and 5) (extracted from the layer “EU Hydro” of Copernicus)	Landscape (1 km)
	Dfarm	Distance (m) from the nearest mink fur farm	Landscape (1 km)
Raft colonization and abandonment probability	All variables from initial probability of raft use		
	S	Primary period	Yearly (Time)
	Catch	Number of mink trapped (during trapping campaigns)	Yearly
	Trap	Trapping attempted (yes/no)	Yearly
Detection probability	Day	Julian day	Observation covariate (Time)
	Month	Month	Observation covariate (Time)
	Year	Year	Observation covariate (Time)
	Session	Primary period	Observation covariate (Time)

### Analysis framework and dynamic occupancy models

We used multi-season dynamic occupancy models to investigate patterns of mink raft use after accounting for imperfect detection^39^. However, for these models to apply, three main assumptions must be met^39^ which are clarified below. According to the first assumption, there should be no false positive detections of the target species. In this study, all animal tracks were photographed and sent to experts for identification and hence false positive detections of minks should not be considered an issue. Based on the second assumption, sample units (rafts) are assumed to be independent. Since the study focused on mink tracks detection rather than detection of the species itself, the detection probability of tracks is independent on whether minks are in close proximity to the raft locations or not, and thus the second assumption could be considered true. Finally, according to the third assumption the status of a sampling unit (raft) should remain unchanged during primary periods^40^. In our case, the terms “occupancy” and “extinction” need to be clarified^41–43^. Occupancy estimates should be interpreted in terms of rafts used by the species; that is rafts where mink were present at some point during or prior to the survey period, as opposed to rafts exclusively inhabited during the survey period. Additionally, since disappearance from a raft implies abandonment rather than local extinction, the probability of extinction should be interpreted as probability of raft abandonment. Thus, we hereafter use the terms “use” instead of “occupancy” and “abandonment” instead of “extinction”.

Based on the above, we modelled the probability of raft use for the first primary period (*ψ*_1_), the probability of raft colonization (*γ*; i.e. the probability of an unused raft at time t becoming used at time t + 1) and raft abandonment (*ε*), and mink detection probability (*p*). In order to reduce the effect of uninformative parameters^44^, we developed a limited number of candidate models following a hierarchical four-stage approach^45^. First, we modelled *p* through a set of models that included each of the observation-level covariates (Table 1), as well as a null model in which no covariates were included (Supplementary Table S1). Second, we used the top ranked *p* model to develop a set of models examining *ψ*_1_ for each spatial scale (Supplementary Tables S2–S4). We then used the covariates included in the top ranked model from each spatial scale to develop a new list of candidate models for *ψ*_1_ (Supplementary Table S5). Third, we used the top ranked *ψ*_1_ model from the previous step to construct a list of candidate models examining *γ* for each spatial scale and for a set of yearly covariates that included the effect of primary period (S), attempted trapping (trap), number of individuals trapped (catch), as well as a null model (Supplementary Tables S6–S9). We then used the covariates from the above top ranked models to create a new list of candidate models for *γ* (Supplementary Table S10). Finally, we used the top ranked *γ* model from the previous step to create a set of models examining *ε* for each spatial scale and for the set of available yearly covariates (Supplementary Tables S11–S14). Using the covariates of the above top ranked models, we created a new list of candidate models for *ε* (Supplementary Table S15). The top ranked model from this step was the selected as the final model on which the interpretation of our results was based. Model fit of the final model was tested using a parametric bootstrap goodness-of-fit test based on Pearson’s χ^2^, where *P* > 0.05 indicates adequate model fit^46,47^. All statistical analyses were carried out in R^48^, with the function *colext* in the package *unmarked*^46^.

## Results

**Figure 2:**
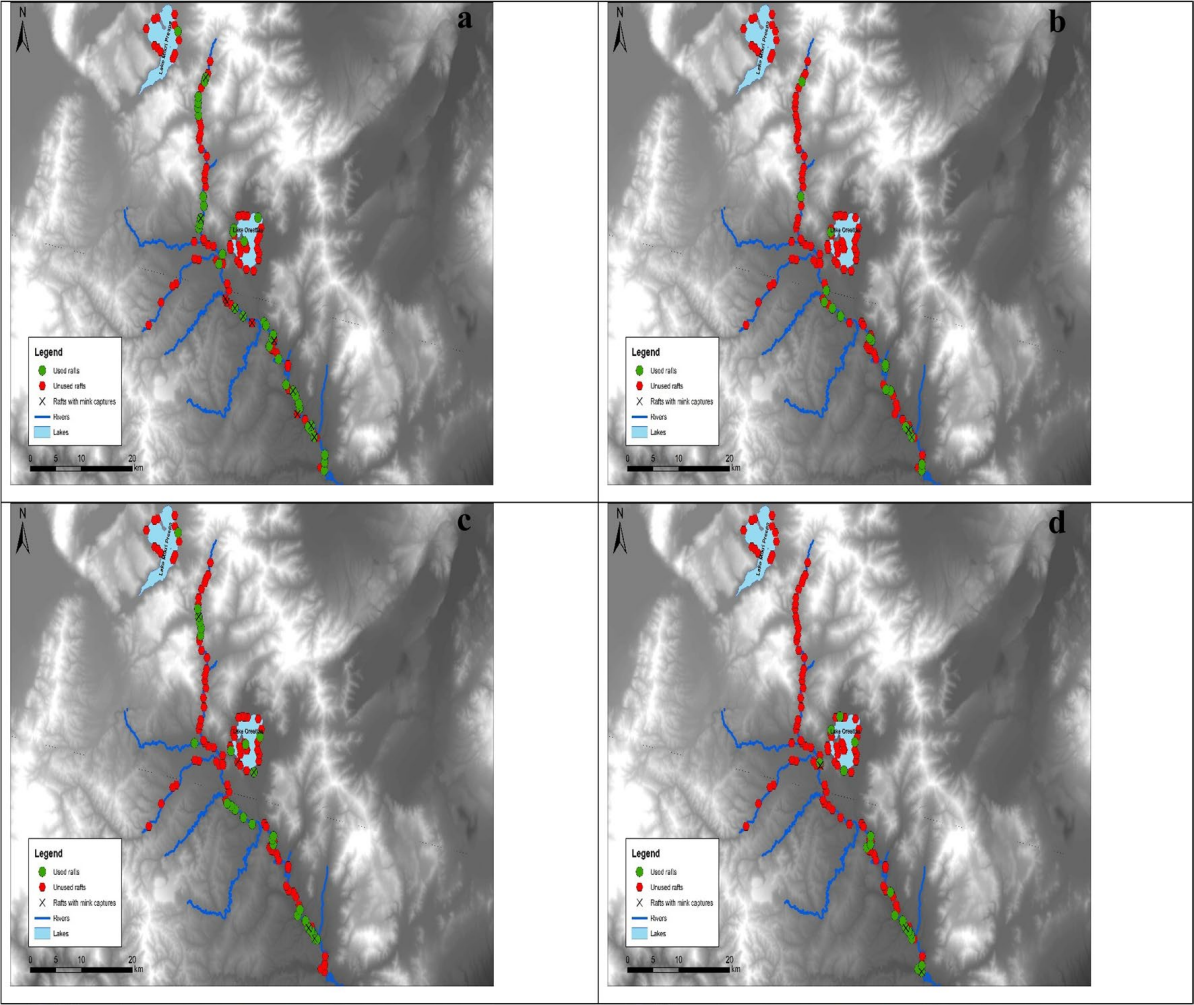
Location of used and unused floating rafts, as well as rafts with American mink captures during (**a**) the first, (**b**) the second, (**c**) the third, and (**d**) the fourth primary period that took place in western Macedonia, Greece, 2021–2023.

Throughout the duration of the study, mink tracks were recorded at least once at 65 floating rafts, whereas a total of 28 individuals were trapped and culled. More specifically, mink tracks were recorded at 38 rafts and 18 individuals were trapped during the first primary period. During the second period mink tracks were recorded at 18 rafts and two individuals were trapped, followed by 27 rafts with mink tracks and five trapped individuals during the third period. Finally, during the fourth and final period, mink tracks were recorded at 18 rafts and three individuals were caught. The rafts used by the species during each period, as well as the rafts in which individuals were trapped are shown in Fig. 2a–d.

**Figure 3:**
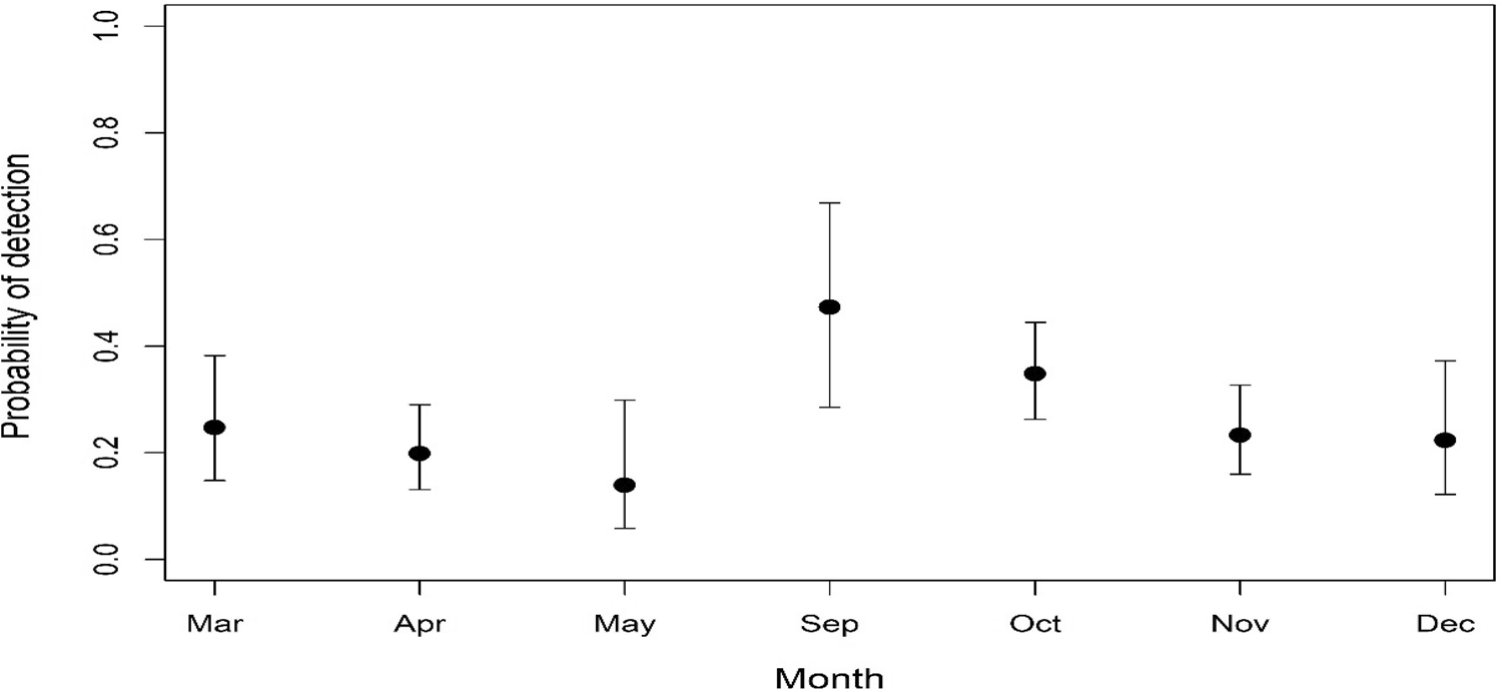
Predicted relationship between survey months and detection probabilities (± 95% CI) of American mink in western Macedonia, Greece, 2021–2023.

**Figure 4:**
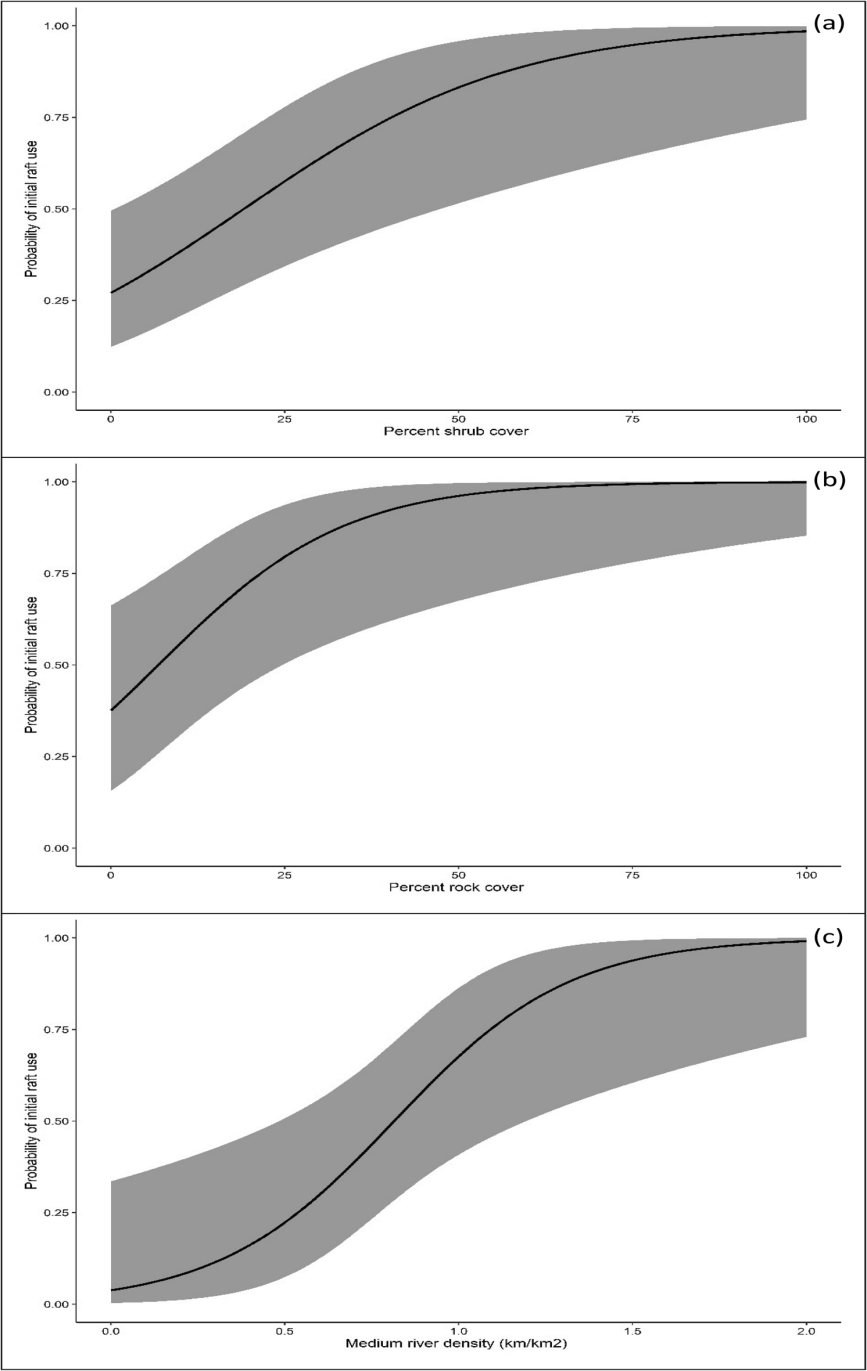
Predicted relationships between (**a**) shrub cover, (**b**) rock cover, and (**c**) medium river density and the probabilities of initial raft use by the American mink in western Macedonia, Greece, 2021–2023. Shaded areas represent confidence intervals (95%) of the estimations.

**Figure 5:**
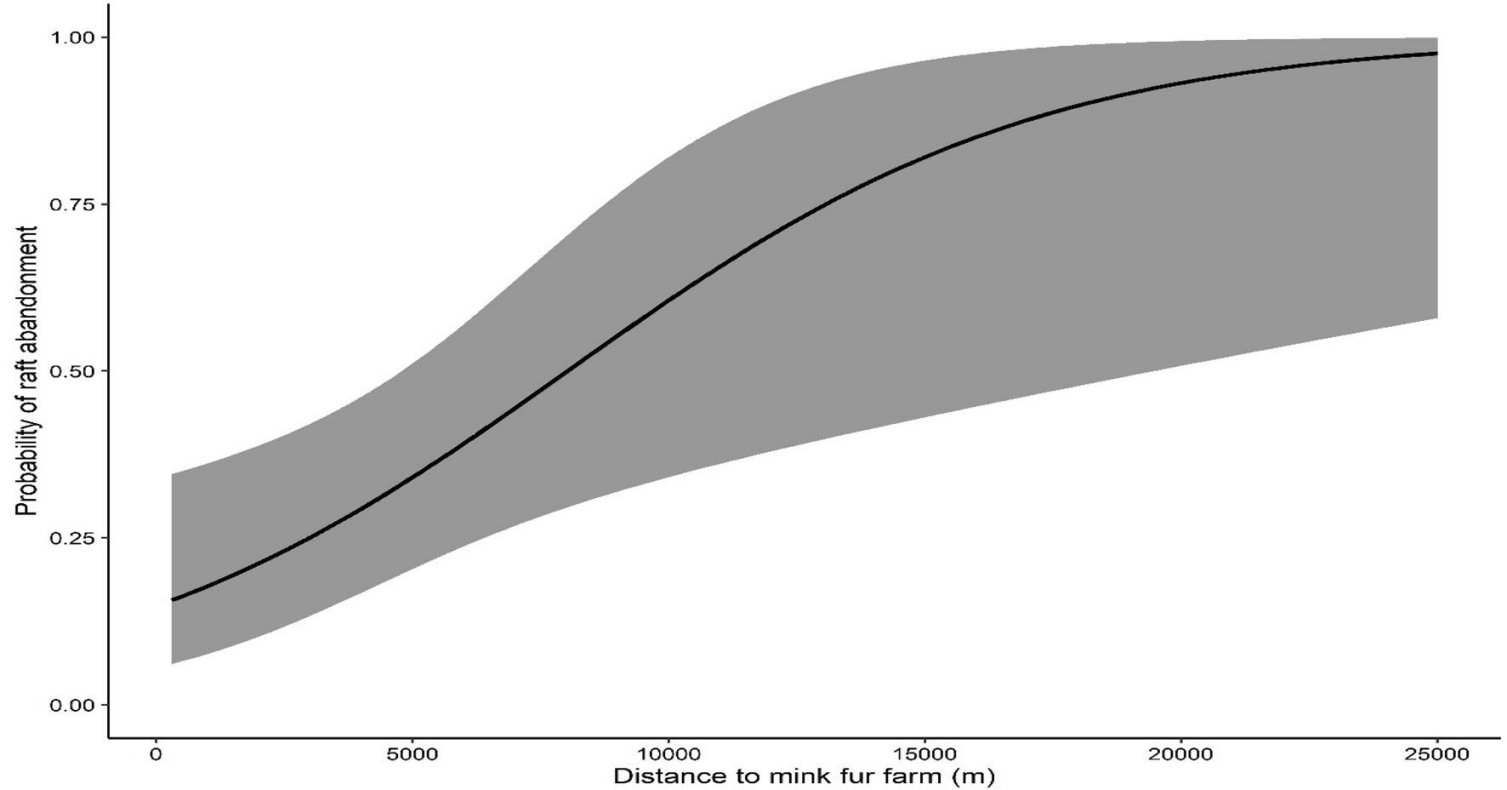
Predicted relationship between distance to the nearest fur farm and probability of local raft abandonment by the American mink in western Macedonia, Greece, 2021–2023. Shaded areas represent confidence intervals (95%) of the estimations.

Based on the results of the final model (Table 2), American mink detectability was influenced by the month during which surveys were conducted. More specifically, detectability was greater during the months of September ($\hat{\beta }$ = 1.287, S.E. = 0.487) and October ($\hat{\beta }$ = 0.768, S.E. = 0.323), whereas during April, detectability was the lowest ($\hat{\beta }$ = -1.395, S.E. = 0.255). Detection probabilities for each month are shown in Fig. 3. The percent amount of shrub ($\hat{\beta }$ = 1.726, S.E. = 0.587) and rock cover ($\hat{\beta }$ = 1.244, S.E. = 0.481) at the micro-habitat scale, as well as the density of medium-sized rivers ($\hat{\beta }$ = 1.985, S.E. = 0.762) at the landscape scale, were found to have a positive effect on the probability of a raft being used by the species (Fig. 4a, b, c). Confidence intervals of all three variables did not span zero, suggesting that they were highly informative (Table 2). Conversely, the probability of a raft being colonized by the species increased with increasing cover of shrubs and reeds at the micro-habitat scale; however, both these variables exhibited wide confidence intervals (Table 2), suggesting that the probability of raft colonization was not well predicted at higher values of shrub and reed cover. Finally, the probability of a raft being abandoned by the species was positively associated with the distance to the nearest mink fur farm ($\hat{\beta }$ = 1.345, S.E. = 0.52), with longer distances resulting in greater probabilities of raft abandonment (Fig. 5). Confidence intervals for this variable did not span zero (Table 2), suggesting that the probability of raft abandonment was well predicted at longer distances from fur farms. The final multi-season occupancy model from which the results were drawn had an adequate model fit according to Pearson’s χ^2^ (*P* = 0.110).

**Table 2:** Results of the final multi-season occupancy model for the American mink in western Macedonia, Greece.

Probability of	Variable	$\hat{\beta }$*	SE
Detection	Month		
	*Intercept* (*Apr*)	− 1.395	0.255
	Dec	0.147	0.442
	Mar	0.281	0.367
	May	− 0.426	0.536
	Nov	0.205	0.339
	***Oct***	0.768	0.323
	***Sep***	1.287	0.487
Initial raft use	*Intercept*	0.735	0.565
	**SH**	1.726	0.587
	**R**	1.244	0.481
	**LRiv1000**	1.985	0.762
Raft colonization	*Intercept*	− 16.6	31.3
	SH	32.4	57.4
	RE	14.7	27.2
Raft abandonment	*Intercept*	− 0.339	0.381
	**Dfarm**	1.345	0.520

Estimates ($\hat{\beta }$) include probability of detection (*p*), initial probability of raft use (*ψ*_*1*_), probability of raft colonization (*γ*), and probability of raft abandonment (*ε*).

*Estimates reported in logit-scale.

Bold fonts indicate variables with 95% confidence intervals not spanning zero.

## Discussion

The ability of invasive species to successfully spread and establish in an area depends on their spatial and temporal responses to environmental features^49^. Occupancy models offer a great opportunity to estimate the factors that determine an invasive species’ presence in space and time, and their outputs can be utilized in designing and guiding management actions^21,50–52^.

Floating rafts constitute one of the most widespread methods for surveying American mink presence in riparian and coastal areas^35,36,53–55^. According to Reynolds et al.^34^ and Reynolds et al.^56^, floating rafts are much more efficient at tracking American mink than alternative methods; however, detection may vary by season. Indeed, the probability of detecting mink in our study area varied by month, with September and October exhibiting the highest probabilities. During this period, juvenile individuals disperse, and animals re-settle in territories^57^, whereas in late October, populations are generally stable^58^, which might explain the higher detection estimates. Similarly, Roy et al.^59^ reported a higher rate of trapped mink during this time. Conversely, individuals were less likely to be detected during the months of April and May. These months correspond to the gestation, parturition and weaning periods, during which mink activity is at its lowest^60,61^. When accounting for imperfect detection, mink occupancy within our study area was estimated to be 67.59%.

Initial use of rafts by the American mink within the study area was found to be mainly driven by features of its immediate habitat. This may reflect the species’ opportunistic behaviour or may be due to its small ranging ability^60,62^. Based on our results, mink was more likely to use rafts established along riverbanks and lake shores with more shrubs and rocks. These patterns have been previously described in several other studies^22,23,25–27,62,63^ and may reflect the species’ requirements in nesting and in avoiding predation or competition^24,25,64–66^. Yamaguchi et al.^25^ and Schüttler et al.^26^ found that the species avoids exposed areas and mainly uses areas with dense vegetation, which provide more cover and nesting opportunities^57,64^. In addition, the species may favour such areas because they offer more feeding opportunities^27^. According to Torre and Diaz^67^ and Torre et al.^68^, in Mediterranean ecosystems, shrubs support a greater diversity and density of small mammals, many of which may serve as prey for the mink. Similarly, rocky areas can be used for denning when the availability of other nesting types is limited^63^ or to avoid predation^63,69^. According to Brainerd et al.^70^, Zalewski^71^, Achterberg et al.^72^ and Stier^73^, mustelids usually favour tree cavities for nesting. However, when availability is low, the species may seek alternative sites, such as areas with rocks or boulders^63^. Elevated rock cavities resemble trees, as both are inaccessible to terrestrial predators^69^, whereas dens located on the ground between rocks provide protection against predators with digging abilities^63^. Hence, predation risk is likely to be a significant factor influencing the choice of cover and den sites for the species^25,57,74,75^. Because many of the minks’ natural predators and competitors can be found across our study area, individuals may be forced to select densely vegetated or rocky riparian areas^6,25,57,74^.

In addition, the probability of initial raft use by the species was found to be positively associated with the density of medium-sized rivers at the landscape scale. Zabala et al.^76^ reported that mustelids show a preference for secondary rivers with sufficient riparian vegetation cover, while they avoid areas with polluted waters or modified riverbeds. Moreover, Sidorovich and Macdonald^18^ reported that mustelids avoid large, fast-flowing rivers, while Garcia et al.^27^ concluded that because secondary rivers are characterized by the existence of islets within the watershed, these may serve as resting sites for the species^25,74^. Our study area does not include large rivers (Stahler order > 6); therefore, the positive association between mink raft use and medium-sized rivers may be because this river category is characterized by continuous water flow throughout the year, in contrast to smaller rivers in the area, which are characterized by seasonal flow and shallow water depth.

The probability of a vacant raft being colonized (used) between surveys was found to be positively associated with the percent cover of shrubs and reeds. However, none of these variables were informative. Previous studies have shown that colonization may be affected by stream size, water depth and flow regime^55,77,78^. In addition, colonization may be closely related to habitat features that increase denning and resting site availability^78^ or may be closely related to prey distribution and urbanization^79^. Conversely, the distance to the nearest fur farm within our study area was very informative in predicting the probability of raft abandonment by the species. Moreover, almost 70% of all mink presence sites were within a 5 km radius from nearby fur farms, whereas 90% of all individuals captured were also within a 5 km radius from nearby farms. According to Bonesi and Palazón^7^, fur farms are considered the main source of American mink reintroduction. With that in mind and given that mink are very elusive species^80^, eradication campaigns are often unsuccessful. The number of minks escaping from fur farms located within the study area is unknown; however, reducing the number of individuals introduced into the wild may help control the successful establishment and spread of American minks in the wider region^81^. Nonetheless, both wild and introduced individuals illustrate similar feeding habits and exhibit the same ability to hunt their prey^82^. In addition, individuals introduced from fur farms may act as a source of increasing genetic diversity and adaptation for wild populations^83^.

## Conclusions

Knowledge of the environmental parameters that influence the occupancy dynamics of invasive species is fundamental in planning and implementing effective management actions^84^. Management options to mitigate the negative impacts caused by American mink introduction and establishment may include direct population control (e.g., removal trapping) or habitat modification^8^. However, managing the species at a small spatial scale is likely to be more practical and effective than managing it at the landscape level^57,85^. In addition, management actions should consider identifying target areas, both for efficiency and economy but also for protecting biodiversity^8^. According to our results, management actions such as trapping American mink individuals could be more effective if they are focused in areas within a 5 km radius from the nearest fur farms and along rocky or densely vegetated riverbanks or lake shores. In addition, trapping efforts during autumn are likely to be more effective since during this time, the species has a high detection probability. On the other hand, mink control may involve habitat management by reducing the suitability of habitats used by the species for nesting and hiding and improving the suitability of habitats for other competing species, such as the otter (*Lutra lutra*). However, in this case, and due to the opportunistic behaviour and the species’ ability to adapt to various conditions and environments, such actions may be more difficult to implement^8^. While our study focuses on mink raft use rather than occupancy, we believe that our findings are of great importance to managers, since floating rafts are among the main methods used to record, monitor, and control the American mink. Future studies should focus on investigating the factors that may affect the species’ ability to colonize new areas, such as water quality^78^ and prey distribution^29^, which were not addressed in the current study but have been shown to be of great importance. Finally, the composition of free-ranging individuals should be investigated in future studies in order to distinguish between wild-born and farm-born individuals. In the latter case, and if most individuals are a product of recent escapes, management measures should focus on maximizing fur farm security and establishing an early warning and rapid eradication system.

### Supplementary Information

Supplementary Tables.

## Data Availability

The datasets analysed during the current study are available from the corresponding author (D.E.B.) upon reasonable request.

## References

[1] Hulme PE, Pyšek P, Nentwig W, Vilà M (2009). Will threat of biological invasions unite the European Union?. Science.

[2] Tittensor DP (2014). A mid-term analysis of progress toward international biodiversity targets. Science.

[3] Blackburn TM, Dyer E, Su S, Cassey P (2015). Long after the event, or four things we (should) know about bird invasions. J. Ornithol..

[4] Vitousek PM, D’antonio CM, Loope LL, Rejmanek M, Westbrooks R (1997). Introduced species: A significant component of human-caused global change. N. Z. J. Ecol..

[5] Sala OE (2000). Global biodiversity scenarios for the year 2100. Science.

[6] Larivière S (1999). Mustela vison. Mamm. Spec..

[7] Bonesi L, Palazon S (2007). The American mink in Europe: Status, impacts, and control. Biol. Conserv..

[8] Macdonald DW, Harrington LA (2003). The American mink: The triumph and tragedy of adaptation out of context. N. Z. J. Zool..

[9] Semos N, Dotas V, Bampidis V (2021). Development strategies for the fur farming industry in Greece. Open J. Bus. Manag..

[10] Henriksen BIF, Møller SH, Malmkvist J (2022). Animal welfare measured at mink farms in Europe. Appl. Anim. Behav. Sci..

[11] Rushton SP, Barreto GW, Cormack RM, Macdonald DW, Fuller R (2000). Modelling the effects of mink and habitat fragmentation on the water vole. J. Appl. Ecol..

[12] Põdra M, Gómez A, Palazón S (2013). Do American mink kill European mink? Cautionary message for future recovery efforts. Eur. J. Wildl. Res..

[13] Põdra M, Gómez A (2018). Rapid expansion of the American mink poses a serious threat to the European mink in Spain. Mammalia.

[14] Mori E, Mazza G (2019). Diet of a semiaquatic invasive mammal in northern Italy: Could it be an alarming threat to the endemic water vole?. Mamm. Biol..

[15] Brzeziński M, Żmihorski M, Nieoczym M, Wilniewczyc P, Zalewski A (2020). The expansion wave of an invasive predator leaves declining waterbird populations behind. Divers. Distrib..

[16] Ferreras P, Macdonald DW (1999). The impact of American mink *Mustela vison* on water birds in the upper Thames. J. Appl. Ecol..

[17] Hammershøj M, Thomsen EA, Madsen AB (2004). Diet of free-ranging American mink and European polecat in Denmark. Acta Theriol. (Warsz).

[18] Sidorovich V, Macdonald DW (2001). Density dynamics and changes in habitat use by the European mink and other native mustelids in connection with the American mink expansion in Belarus. Neth. J. Zool..

[19] Manas S, Cena JC, Ruiz-Olmo J, Palazón S, Domingo M, Wolfinbarger JB, Bloom ME (2001). Aleutian mink disease parvovirus in wild riparian carnivores in Spain. J. Wildl. Dis..

[20] Vada R, Illanas S, Acevedo P, Adriaens T, Apollonio M, Belova O, Blanco-Aguiar JA, Csányi S, Body G, Fernández-De-Mera IG, Ferroglio E (2023). Feral American mink *Neogale** vison* continues to expand its European range: Time to harmonise population monitoring and coordinate control. Mamm. Rev..

[21] Crego RD, Jimenez JE, Rozzi R (2018). Potential niche expansion of the American mink invading a remote island free of native-predatory mammals. PLoS One.

[22] Allen, A. W. Western Energy and Land Use Team, Division of Biological Services, Research and Development, Fish and Wildlife Service, US Department of the Interior. *Habitat Suitability Index Models: Mink* (1984).

[23] Previtali A, Cassini MH, Macdonald DW (1998). Habitat use and diet of the American mink (*Mustela vison*) in Argentinian Patagonia. J Zool.

[24] Bonesi L, Dunstone N, O’Connell M (2000). Winter selection of habitats within intertidal foraging areas by mink (*Mustela vison*). J. Zool..

[25] Yamaguchi N, Rushton S, Macdonald DW (2003). Habitat preferences of feral American mink in the Upper Thames. J. Mammal..

[26] Schüttler E, Ibarra JT, Gruber B, Rozzi R, Jax K (2010). Abundance and habitat preferences of the southernmost population of mink: Implications for managing a recent island invasion. Biodivers. Conserv..

[27] García P, Arévalo V, Lizana M (2010). Characterisation of den sites of American mink Neovison vison in central Spain. Wildl. Biol..

[28] Carlsson NOL, Jeschke JM, Holmqvist N, Kindberg J (2010). Long-term data on invaders: When the fox is away, the mink will play. Biol. Invasions.

[29] Wolff PJ, Taylor CA, Heske EJ, Schooley RL (2015). Habitat selection by American mink during summer is related to hotspots of crayfish prey. Wildl. Biol..

[30] Hodder DP, Larsen KW, Crowley SM (2017). The role of environmental variables and sympatric meso-carnivores on the detection and occupancy of American mink during winter. Hystrix.

[31] Galanaki A, Kominos T (2022). The distribution of American mink (*Neovison vison*) in Greece. Mammalia.

[32] Ministry of Environment—Special Water Secretariat. *River Basin Management Plan of Western Macedonia* (2014).

[33] Ministry of Environment—Special Water Secretariat. *Special Management Plan for the Sub-basin of Prespa in the River Basin of Prespa (GR01)* (2014).

[34] Reynolds JC, Short MJ, Leigh RJ (2004). Development of population control strategies for mink *Mustela vison*, using floating rafts as monitors and trap sites. Biol. Conserv..

[35] Porteus T, Short M, Richardson S, Reynolds J (2012). Empirical development of strategy for the control of invasive American mink by trapping. Eur. J. Wildl. Res..

[36] Reynolds JC, Richardson SM, Rodgers BJE, Rodgers ORK (2013). Effective control of non-native American mink by strategic trapping in a river catchment in mainland Britain. J. Wildl. Manage..

[37] Melero Y, Palazón S, Revilla E, Martelo J, Gosálbez J (2008). Space use and habitat preferences of the invasive American mink (*Mustela vison*) in a Mediterranean area. Eur. J. Wildl. Res..

[38] ESRI (2011). Desktop Release 10.

[39] MacKenzie DI, Nichols JD, Hines JE, Knutson MG, Franklin AB (2003). Estimating site occupancy, colonization, and local extinction when a species is detected imperfectly. Ecology.

[40] Rota CT, Fletcher RJ, Dorazio RM, Betts MG (2009). Occupancy estimation and the closure assumption. J. Appl. Ecol..

[41] Dagtekin D, Ertürk A, Sommer S, Ozgul A, Soyumert A (2024). Seasonal habitat-use patterns of large mammals in a human-dominated landscape. J. Mammal..

[42] Louvrier J (2018). Mapping and explaining wolf recolonization in France using dynamic occupancy models and opportunistic data. Ecography.

[43] Roda F (2021). Wolf scat detection dog improves wolf genetic monitoring in new French colonized areas. J. Vertebr. Biol..

[44] Arnold TW (2010). Uninformative parameters and model selection using Akaike’s Information Criterion. J. Wildl. Manage..

[45] Ramírez-Cruz GA, Ortega-Álvarez R (2021). Identifying management guidelines to control the invasive House Sparrow (*Passer*
*domesticus*) within natural protected areas through the estimation of local colonization and extinction probabilities. Biol. Invasions.

[46] Fiske I, Chandler R (2011). Unmarked: An R package for fitting hierarchical models of wildlife occurrence and abundance. J. Stat. Softw..

[47] Kéry, M. & Chandler, R. Dynamic occupancy models in unmarked (2012). http://cran.r-project.org/web/packages/unmarked/vignettes/colext.pdf Accessed 20 April 2015

[48] R Core Team, R. R: A language and environment for statistical computing (2013).

[49] Catford JA, Jansson R, Nilsson C (2009). Reducing redundancy in invasion ecology by integrating hypotheses into a single theoretical framework. Divers. Distrib..

[50] Bled F, Royle JA, Cam E (2011). Hierarchical modeling of an invasive spread: The Eurasian collared-dove *Streptopelia*
*decaocto* in the United States. Ecol. Appl..

[51] Yackulic CB (2012). Neighborhood and habitat effects on vital rates: Expansion of the Barred owl in the Oregon coast ranges. Ecology.

[52] Santulli G, Palazón S, Melero Y, Gosálbez J, Lambin X (2014). Multi-season occupancy analysis reveals large scale competitive exclusion of the critically endangered European mink by the invasive non-native American mink in Spain. Biol. Conserv..

[53] Harrington LA, Harrington AL, Macdonald DW (2008). Estimating the relative abundance of American mink *Mustela vison* on lowland rivers: evaluation and comparison of two techniques. Eur. J. Wildl. Res..

[54] Bryce R (2011). Turning back the tide of American mink invasion at an unprecedented scale through community participation and adaptive management. Biol. Conserv..

[55] Schooley RL, Cotner LA, Ahlers AA, Heske EJ, Levengood JM (2012). Monitoring site occupancy for American mink in its native range. J. Wildl. Manage..

[56] Reynolds JC, Porteus TA, Richardson SM, Leigh RJ, Short MJ (2010). Detectability of American mink using rafts to solicit field signs in a population control context. J. Wildl. Manage..

[57] Dunstone N (1993). The Mink.

[58] Yamaguchi N, Macdonald DW (2003). The burden of co-occupancy: Intraspecific resource competition and spacing patterns in American mink, *Mustela vison*. J. Mammal..

[59] Roy, S., Reid, N. & McDonald, R. A. A review of mink predation and control in Ireland. in *Irish Wildlife Manuals* vol. 40 (National Parks and Wildlife Service, Department of the Environment, Heritage and Local Government, 2009).

[60] Dunstone N, Birks JDS (1985). The comparative ecology of coastal, riverine and lacustrine mink Mustela vison in Britain. Zeitschrift für Angew. Zool..

[61] Bonesi L, Macdonald DW (2004). Evaluation of sign surveys as a way to estimate the relative abundance of American mink (*Mustela vison*). J. Zool..

[62] Lundy MG, Montgomery WI (2010). A multi-scale analysis of the habitat associations of European otter and American mink and the implications for farm scale conservation schemes. Biodivers. Conserv..

[63] Birks JDS, Messenger JE, Halliwell EC (2005). Diversity of den sites used by pine martens *Martes*
*martes*: A response to the scarcity of arboreal cavities?. Mamm. Rev..

[64] Halliwell EC, Macdonald DW (1996). American mink *Mustela*
*vison* in the Upper Thames catchment: Relationship with selected prey species and den availability. Biol. Conserv..

[65] McDonald RA (2002). Resource partitioning among British and Irish mustelids. J. Anim. Ecol..

[66] Bonesi L, Harrington LA, Maran T, Sidorovich VE, Macdonald DW (2006). Demography of three populations of American mink *Mustela*
*vison* in Europe. Mamm. Rev..

[67] Torre I, Díaz M (2004). Small mammal abundance in Mediterranean post-fire habitats: A role for predators?. Acta Oecol..

[68] Torre I, Jaime-González C, Díaz M (2022). Habitat suitability for small mammals in Mediterranean landscapes: How and why shrubs matter. Sustainability.

[69] Webster JA (2001). A review of the historical evidence of the habitat of the pine marten in Cumbria. Mamm. Rev..

[70] Brainerd SM, Helldin JO, Lindstrom ER, Rolstad E, Rolstad J, Storch I (1995). Pine marten (*Martes*
*martes*) selection of resting and denning sites in Scandinavian managed forests. Ann. Zool. Fennici..

[71] Zalewski A (1997). Factors affecting selection of resting site type by pine marten in primeval deciduous forests (Bialowieza National Park, Poland). Acta Theriol. (Warsz).

[72] Achterberg C, Bestman M, Wijsman HJW (2000). Inventarisatie van boommarternestbomen op de Utrechtse Heuvelrug 1992–1999. Lutra.

[73] Stier N (2000). Tagesverstecke des Baummarders (*Martes martes* L.) in Südwest-Mecklenburg. Beiträge zur Jagd-und Wildforschung.

[74] Zabala J, Zuberogoitia I, Martínez-Climent JA (2007). Winter habitat preferences of feral American mink *Mustela vison* in Biscay, Northern Iberian Peninsula. Acta Theriol. (Warsz).

[75] Harrington LA, Macdonald DW (2008). Spatial and temporal relationships between invasive American mink and native European polecats in the southern United Kingdom. J. Mammal..

[76] Zabala J, Zuberogoitia I, Martínez-Climent JA (2006). Factors affecting occupancy by the European mink in south-western Europe/Facteurs affectant l’habitat du vison en Europe sud-occidentale. Mammalia.

[77] Ahlers AA (2015). Summer precipitation predicts spatial distributions of semiaquatic mammals. PLoS One.

[78] Holland AM, Schauber EM, Nielsen CK, Hellgren EC (2019). River otter and mink occupancy dynamics in riparian systems. J. Wildl. Manage..

[79] Ahlers AA, Heske EJ, Schooley RL (2016). Prey distribution, potential landscape supplementation, and urbanization affect occupancy dynamics of American mink in streams. Landsc. Ecol..

[80] Medina-Vogel G, Barros M, Monsalve R, Pons DJ (2015). Assessment of the efficiency in trapping North American mink (*Neovison vison*) for population control in Patagonia. Rev. Chile. Historia Nat..

[81] Jeschke JM, Strayer DL (2006). Determinants of vertebrate invasion success in Europe and North America. Glob. Change Biol..

[82] Rørbæk RW, Andersen TA, Pertoldi C, Jørgensen A, Pagh S (2023). Diet of free ranging American mink (*Neovison vison*) in Denmark. Animals.

[83] Pagh S (2019). Methods for the identification of farm escapees in feral mink (*Neovison vison*) populations. PLoS One.

[84] MacKenzie DI, Nichols JD, Royle JA, Pollock KH, Bailey L, Hines JE (2017). Occupancy Estimation And modeling: Inferring Patterns and Dynamics of Species Occurrence.

[85] Boitani, L. Carnivore introductions and invasions: Their success and management. In *Carnivore Conservation*, 123–144 (1999).

