# Supplementary material for: Occupancy dynamics of free ranging American mink (Neogale vison) in Greece
Source: Sci Rep. 2024 Apr 30;14:9973. doi: 10.1038/s41598-024-60542-4 (PMC11063139; doi:10.1038/s41598-024-60542-4)
Supplement: Supplementary file 1 — Supplementary Tables. [file 41598_2024_60542_MOESM1_ESM.pdf]

Supplementary Table S1. Model results for probability of detection (*p*) from multi-season occupancy models of American mink in western Macedonia, Greece, 2021-2023. Occupancy (Ψ), colonization (γ), and extinction (ε) were held constant in all models. Models are ranked based on the lowest Akaike's Information Criterion (*AIC*) where *deltaAIC* = AIC_i – minimum AIC, *nPars* = number of parameters, and *AICwt* = AIC weight.

Model	AIC	deltaAIC	nPars	AICwt
**Ψ(.) γ(.) ε(.) *p*(month)**	**798,77**	**0,00**	**10**	**0,47**
Ψ(.) γ(.) ε(.) *p*(session)	799,47	0,69	7	0,32
Ψ(.) γ(.) ε(.) *p*(julian day)	801,92	3,14	5	0,1
Ψ(.) γ(.) ε(.) *p*(null)	802,14	3,37	4	0,09
Ψ(.) γ(.) ε(.) *p*(year)	804,79	6,02	5	0,02

Supplementary Table S2. Model results for probability of occupancy (ψ) from multi-season occupancy models of American mink in western Macedonia, Greece, 2021-2023 based on micro-habitat variables. Models included top detection model (month), represented as *p*(top). Colonization (γ), and extinction (ε) were held constant in all models. Models are ranked based on the lowest Akaike's Information Criterion (*AIC*) where *deltaAIC* = AICi – minimum AIC, *nPars* = number of parameters, and *AICwt* = AIC weight.

Model	AIC	deltaAIC	nPars	AICwt
**Ψ(SH+RE+R) γ(.) ε(.) *p*(top)**	**750,57**	**0,00**	**13**	**0,41**
Ψ(SH+R) γ(.) ε(.) *p*(top)	751,46	0,89	12	0,26
Ψ(SH+RE+DEM) γ(.) ε(.) *p*(top)	753,92	3,35	13	0,08
Ψ(SH+Strata3) γ(.) ε(.)*p*(top)	754,26	3,68	12	0,07
Ψ(SH+DEM) γ(.) ε(.)*p*(top)	754,68	4,11	12	0,05
Ψ(SH) γ(.) ε(.)*p*(top)	754,90	4,32	11	0,05
Ψ(SH+RE) γ(.) ε(.)*p*(top)	754,90	4,33	12	0,04
Ψ(SH+RE+Strata3) γ(.) ε(.) *p*(top)	755,45	4,88	13	0,04
Ψ(RE+Strata3+DEM) γ(.) ε(.) *p*(top)	771,76	21,19	13	0,00
Ψ(RE+DEM) γ(.) ε(.) *p*(top)	772,28	21,70	12	0,00
Ψ(RE+Strata3+R) γ(.) ε(.) *p*(top)	777,60	27,03	13	0,00
Ψ(RE+R) γ(.) ε(.) *p*(top)	779,11	28,54	12	0,00
Ψ(RE+Strata3) γ(.) ε(.) *p*(top)	779,37	28,80	12	0,00
Ψ(RE) γ(.) ε(.) *p*(top)	781,37	30,80	11	0,00
Ψ(Strata3) γ(.) ε(.) *p*(top)	784,85	34,27	11	0,00
Ψ(Strata3+R) γ(.) ε(.) *p*(top)	785,89	35,31	12	0,00
Ψ(DEM+R) γ(.) ε(.) *p*(top)	786,52	35,94	12	0,00
Ψ(DEM) γ(.) ε(.) *p*(top)	787,31	36,74	11	0,00
Ψ(Strata2) γ(.) ε(.) *p*(top)	791,74	41,17	11	0,00
Ψ(GS) γ(.) ε(.) *p*(top)	796,64	46,07	11	0,00
Ψ(R) γ(.) ε(.) *p*(top)	798,16	47,58	11	0,00
Ψ(null) γ(.) ε(.) *p*(top)	798,77	48,20	10	0,00
Ψ(BS) γ(.) ε(.) *p*(top)	799,53	48,96	11	0,00

Supplementary Table S3. Model results for probability of occupancy (ψ) from multi-season occupancy models of American mink in western Macedonia, Greece, 2021-2023 based on local 250 m. variables. Models included top detection model (month), represented as *p*(top). Colonization (γ), and extinction (ε) were held constant in all models. Models are ranked based on the lowest Akaike's Information Criterion (*AIC*) where *deltaAIC* = AICi – minimum AIC, *nPars* = number of parameters, and *AICwt* = AIC weight.

Model	AIC	deltaAIC	nPars	AICwt
**Ψ(Broad250+HetAgri250+Arable250) γ(.) ε(.) *p*(top)**	**789,56**	**0,00**	**13**	**0,39**
Ψ(Broad250+HetAgri250) γ(.) ε(.) *p*(top)	790,53	0,97	12	0,23
Ψ(Broad250+Riv250+HetAgri250) γ(.) ε(.) *p*(top)	791,95	2,40	13	0,12
Ψ(Broad250+Riv250) γ(.) ε(.) *p*(top)	792,09	2,53	12	0,11
Ψ(Broad250) γ(.) ε(.) *p*(top)	793,13	3,57	11	0,06
Ψ(Broad250+Arable250) γ(.) ε(.) *p*(top)	793,74	4,18	12	0,05
Ψ(HetAgri250+Arable250) γ(.) ε(.) *p*(top)	797,45	7,89	12	0,01
Ψ(Riv250) γ(.) ε(.) *p*(top)	797,97	8,41	11	0,00
Ψ(HetAgri250) γ(.) ε(.) *p*(top)	798,39	8,83	11	0,01
Ψ(Riv250+Arable250) γ(.) ε(.) *p*(top)	798,58	9,02	12	0,00
Ψ(null) γ(.) ε(.) *p*(top)	798,77	9,22	10	0,01
Ψ(Riv250+HetAgri250+Arable250) γ(.) ε(.) *p*(top)	799,03	9,47	13	0,00
Ψ(Arable250) γ(.) ε(.) *p*(top)	799,05	9,49	11	0,01
Ψ(Riv250+HetAgri250) γ(.) ε(.) *p*(top)	799,23	9,67	12	0,00
Ψ(Shrub250) γ(.) ε(.) *p*(top)	800,02	10,46	11	0,00
Ψ(Dev250) γ(.) ε(.) *p*(top)	800,39	10,83	11	0,00

Supplementary Table S4. Model results for probability of occupancy (ψ) from multi-season occupancy models of American mink in western Macedonia, Greece, 2021-2023 based on landscape 1km variables. Models included top detection model (month), represented as *p*(top). Colonization (γ), and extinction (ε) were held constant in all models. Models are ranked based on the lowest Akaike's Information Criterion (*AIC*) where *deltaAIC* = AICi – minimum AIC, *nPars* = number of parameters, and *AICwt* = AIC weight.

Model	AIC	deltaAIC	nPars	AICwt
**Ψ(Dfarm+LRiv1000) γ(.) ε(.) *p*(top)**	**776,02**	**0,00**	**12**	**0,31**
Ψ(Dfarm+LRiv1000+Dev1000) γ(.) ε(.) *p*(top)	777,44	1,42	13	0,15
Ψ(LRiv1000) γ(.) ε(.) *p*(top)	777,56	1,54	11	0,14
Ψ(LRiv1000+Dev1000) γ(.) ε(.) *p*(top)	777,65	1,63	12	0,13
Ψ(Dfarm+LRiv1000+Shrub1000) γ(.) ε(.) *p*(top)	777,99	1,97	13	0,12
Ψ(LRiv1000+Shrub1000) γ(.) ε(.) *p*(top)	779,43	3,41	12	0,05
Ψ(LRiv1000+Shrub1000+Dev1000) γ(.) ε(.) *p*(top)	779,62	3,60	13	0,05
Ψ(Dfarm) γ(.) ε(.) *p*(top)	780,98	4,96	11	0,03
Ψ(Dfarm+Shrub1000) γ(.) ε(.) *p*(top)	782,77	6,75	12	0,01
Ψ(Dfarm+Dev1000) γ(.) ε(.) *p*(top)	782,85	6,83	12	0,01
Ψ(HetAgri1000) γ(.) ε(.) *p*(top)	791,77	15,75	11	0,00
Ψ(Arable1000) γ(.) ε(.) *p*(top)	794,55	18,53	11	0,00
Ψ(SRiv1000) γ(.) ε(.) *p*(top)	796,47	20,45	11	0,00
Ψ(null) γ(.) ε(.) *p*(top)	798,77	22,75	10	0,00
Ψ(Broad1000) γ(.) ε(.) *p*(top)	799,04	23,02	11	0,00
Ψ(Dev1000) γ(.) ε(.) *p*(top)	800,75	24,73	11	0,00
Ψ(Shrub1000) γ(.) ε(.) *p*(top)	800,77	24,75	11	0,00
Ψ(Shrub1000+Dev1000) γ(.) ε(.) *p*(top)	802,75	26,73	12	0,00

Supplementary Table S5. Model results for probability of occupancy (ψ) from multi-season occupancy models of American mink in western Macedonia, Greece, 2021-2023 based on top models' variables (microhabitat, local 250 m., and landscape 1km). Models included top detection model (month), represented as *p*(top). Colonization (γ), and extinction (ε) were held constant in all models. Models are ranked based on the lowest Akaike's Information Criterion (*AIC*) where *deltaAIC* = AICi – minimum AIC, *nPars* = number of parameters, and *AICwt* = AIC weight.

Model	AIC	deltaAIC	nPars	AICwt
**Ψ(SH+LRiv1000+R) γ(.) ε(.) *p*(top)**	**740,20**	**0,00**	**13**	**0,86**
Ψ(SH+Dfarm) γ(.) ε(.) *p*(top)	746,59	6,39	12	0,03
Ψ(SH+LRiv1000+Dfarm) γ(.) ε(.) *p*(top)	746,60	6,40	13	0,04
Ψ(SH+LRiv1000) γ(.) ε(.) *p*(top)	747,02	6,82	12	0,02
Ψ(SH+LRiv1000+Broad250) γ(.) ε(.) *p*(top)	747,42	7,22	13	0,03
Ψ(SH+LRiv1000+RE) γ(.) ε(.) *p*(top)	748,90	8,70	13	0,01
Ψ(SH+RE+R) γ(.) ε(.) *p*(top)	750,57	10,38	13	0,00
Ψ(SH+Broad250) γ(.) ε(.) *p*(top)	751,40	11,20	12	0,01
Ψ(SH+R) γ(.) ε(.) *p*(top)	751,46	11,27	12	0,00
Ψ(SH) γ(.) ε(.) *p*(top)	754,90	14,70	11	0,00
Ψ(SH+RE) γ(.) ε(.) *p*(top)	754,90	14,71	12	0,00
Ψ(LRiv1000+R) γ(.) ε(.) *p*(top)	770,91	30,71	12	0,00
Ψ(LRiv1000+Dfarm+R) γ(.) ε(.) *p*(top)	771,10	30,91	13	0,00
Ψ(LRiv1000+Dfarm+RE) γ(.) ε(.) *p*(top)	773,93	33,73	13	0,00
Ψ(Dfarm+LRiv1000) γ(.) ε(.) *p*(top)	776,02	35,82	12	0,00
Ψ(LRiv1000+RE) γ(.) ε(.) *p*(top)	776,13	35,94	12	0,00
Ψ(LRiv1000+Dfarm+Broad250) γ(.) ε(.) *p*(top)	776,56	36,36	13	0,00
Ψ(LRiv1000) γ(.) ε(.) *p*(top)	777,56	37,36	11	0,00
Ψ(LRiv1000+Broad250) γ(.) ε(.) *p*(top)	777,58	37,38	12	0,00
Ψ(Dfarm) γ(.) ε(.) *p*(top)	780,98	40,78	11	0,00
Ψ(RE) γ(.) ε(.) *p*(top)	781,37	41,18	11	0,00
Ψ(Broad250+HetAgri250+Arable250) γ(.) ε(.) *p*(top)	789,56	49,36	13	0,00
Ψ(Broad250) γ(.) ε(.) *p*(top)	793,13	52,93	11	0,00
Ψ(R) γ(.) ε(.) *p*(top)	798,16	57,96	11	0,00
Ψ(HetAgri250) γ(.) ε(.) *p*(top)	798,39	58,19	11	0,00
Ψ(null) γ(.) ε(.) *p*(top)	798,77	58,58	10	0,00
Ψ(Arable250) γ(.) ε(.) *p*(top)	799,05	58,85	11	0,00

Supplementary Table S6. Model results for probability of colonization (γ) from multi-season occupancy models of American mink in western Macedonia, Greece, 2021-2023 based on yearly covariates. Models included top detection model (month), represented as *p*(top), and top initial occupancy model (SH+R+LRiv1000) as ψ(top). Colonization (γ), was held constant in all models. Models are ranked based on the lowest Akaike's Information Criterion (*AIC*) where *deltaAIC* = AICi – minimum AIC, *nPars* = number of parameters, and *AICwt* = AIC weight.

Model	AIC	deltaAIC	nPars	AICwt
**Ψ(top) γ(null) ε(.) *p*(top)**	**740,20**	**0,00**	**13**	**0,43**
Ψ(top) γ(S) ε(.) *p*(top)	741,57	1,38	15	0,22
Ψ(top) γ(trap) ε(.) *p*(top)	741,87	1,68	14	0,19
Ψ(top) γ(catch) ε(.) *p*(top)	742,17	1,98	14	0,16

Supplementary Table S7. Model results for probability of colonization (γ) from multi-season occupancy models of American mink in western Macedonia, Greece, 2021-2023 based on microhabitat variables. Models included top detection model (month), represented as *p*(top), and top initial occupancy model (SH+R+LRiv1000) as ψ(top). Colonization (γ), was held constant in all models. Models are ranked based on the lowest Akaike's Information Criterion (*AIC*) where *deltaAIC* = AICi – minimum AIC, *nPars* = number of parameters, and *AICwt* = AIC weight.

Model	AIC	deltaAIC	nPars	AICwt
**Ψ(top) γ(SH+RE) ε(.) *p*(top)**	**729,46**	**0,00**	**15**	**0,78**
Ψ(top) γ(RE+DEM) ε(.) *p*(top)	734,21	4,75	15	0,07
Ψ(top) γ(SH) ε(.) *p*(top)	735,18	5,72	14	0,05
Ψ(top) γ(SH+BS) ε(.) *p*(top)	735.87	6,41	15	0,03
Ψ(top) γ(SH+DEM) ε(.) *p*(top)	736,85	7,39	15	0,02
Ψ(top) γ(BS+DEM) ε(.) *p*(top)	738,33	8,87	15	0,01
Ψ(top) γ(RE) ε(.) *p*(top)	738,88	9,42	14	0,01
Ψ(top) γ(BS) ε(.) *p*(top)	738,93	9,47	14	0,00
Ψ(top) γ(DEM) ε(.) *p*(top)	739,52	10,06	14	0,01
Ψ(top) γ(RE+BS) ε(.) *p*(top)	739,73	10,27	15	0,00
Ψ(top) γ(GS) ε(.) *p*(top)	739,84	10,38	14	0,01
Ψ(top) γ(R) ε(.) *p*(top)	740,17	10,71	14	0,00
Ψ(top) γ(null) ε(.) *p*(top)	740,20	10,74	13	0,01
Ψ(top) γ(Strata2) ε(.) *p*(top)	740,90	11,44	14	0,00
Ψ(top) γ(Strata3) ε(.) *p*(top)	741,17	11,71	14	0,00

Supplementary Table S8. Model results for probability of colonization (γ) from multi-season occupancy models of American mink in western Macedonia, Greece, 2021-2023 based on local 250 m variables. Models included top detection model (month), represented as *p*(top), and top initial occupancy model (SH+R+LRiv1000) as ψ(top). Colonization (γ), was held constant in all models. Models are ranked based on the lowest Akaike's Information Criterion (*AIC*) where *deltaAIC* = AICi – minimum AIC, *nPars* = number of parameters, and *AICwt* = AIC weight.

Model	AIC	deltaAIC	nPars	AICwt
**Ψ(top) γ(Shrub250+Arable250) ε(.) *p*(top)**	**738,07**	**0,00**	**15**	**0,28**
Ψ(top) γ(Shrub250) ε(.) *p*(top)	739,70	1,63	14	0,13
Ψ(top) γ(Dev250) ε(.) *p*(top)	739,98	1,91	14	0,10
Ψ(top) γ(Arable250) ε(.) *p*(top)	740,09	2,02	14	0,11
Ψ(top) γ(null) ε(.) *p*(top)	740,20	2,13	13	0,09
Ψ(top) γ(Shrub250+Dev250) ε(.) *p*(top)	740,61	2,54	15	0,08
Ψ(top) γ(Dev250+Arable250) ε(.) *p*(top)	741,23	3,16	15	0,06
Ψ(top) γ(HetAgri250) ε(.) *p*(top)	741,24	3,17	14	0,06
Ψ(top) γ(Broad250) ε(.) *p*(top)	741,24	3,17	14	0,05
Ψ(top) γ(Riv250) ε(.) *p*(top)	742,20	4,13	14	0,04

Supplementary Table S9. Model results for probability of colonization (γ) from multi-season occupancy models of American mink in western Macedonia, Greece, 2021-2023 based on landscape 1km variables. Models included top detection model (month), represented as *p*(top), and top initial occupancy model (SH+R+LRiv1000) as ψ(top). Colonization (γ), was held constant in all models. Models are ranked based on the lowest Akaike's Information Criterion (*AIC*) where *deltaAIC* = AICi – minimum AIC, *nPars* = number of parameters, and *AICwt* = AIC weight.

Model	AIC	deltaAIC	nPars	AICwt
**Ψ(top) γ(Dfarm+LRiv1000) ε(.) *p*(top)**	**736,65**	**0,00**	**15**	**0,24**
Ψ(top) γ(Dev1000+Shrub1000) ε(.) *p*(top)	737,52	0,87	15	0,15
Ψ(top) γ(Dev1000+Broad1000) ε(.) *p*(top)	738,87	2,22	15	0,08
Ψ(top) γ(Dev1000) ε(.) *p*(top)	739,78	3,13	14	0,05
Ψ(top) γ(null) ε(.) *p*(top)	740,20	3,55	13	0,04
Ψ(top) γ(Arable1000+Shrub1000) ε(.) *p*(top)	740,41	3,77	15	0,04
Ψ(top) γ(Arable1000) ε(.) *p*(top)	740,43	3,78	14	0,04
Ψ(top) γ(Arable1000+Dfarm) ε(.) *p*(top)	740,55	3,90	15	0,03
Ψ(top) γ(Dev1000+Arable1000) ε(.) *p*(top)	740,69	4,04	15	0,03
Ψ(top) γ(Shrub1000) ε(.) *p*(top)	740,91	4,26	14	0,03
Ψ(top) γ(Dfarm) ε(.) *p*(top)	741,01	4,36	14	0,03
Ψ(top) γ(Broad1000) ε(.) *p*(top)	741,29	4,64	14	0,02
Ψ(top) γ(SRiv1000) ε(.) *p*(top)	741,39	4,74	14	0,02
Ψ(top) γ(Dev1000+Dfarm) ε(.) *p*(top)	741,50	4,86	15	0,02
Ψ(top) γ(Arable1000+Broad1000) ε(.) *p*(top)	741,64	4,99	15	0,02
Ψ(top) γ(Shrub1000+Dfarm) ε(.) *p*(top)	741,66	5,01	15	0,02
Ψ(top) γ(Shrub1000+SRiv1000) ε(.) *p*(top)	741,73	5,08	15	0,02
Ψ(top) γ(Shrub1000+HetAgri1000) ε(.) *p*(top)	741,74	5,09	15	0,02
Ψ(top) γ(HetAgri1000) ε(.) *p*(top)	741,84	5,19	14	0,02
Ψ(top) γ(Arable1000+SRiv1000) ε(.) *p*(top)	742,10	5,45	15	0,01
Ψ(top) γ(LRiv1000) ε(.) *p*(top)	742,11	5,46	14	0,02
Ψ(top) γ(Shrub1000+Broad1000) ε(.) *p*(top)	742,32	5,67	15	0,01
Ψ(top) γ(Dfarm+Broad1000) ε(.) *p*(top)	742,50	5,85	15	0,02
Ψ(top) γ(Dfarm+SRiv1000) ε(.) *p*(top)	742,57	5,92	15	0,01
Ψ(top) γ(Dfarm+HetAgri1000) ε(.) *p*(top)	742,74	6,09	15	0,01

Supplementary Table S10. Model results for probability of colonization (γ) from multi-season occupancy models of American mink in western Macedonia, Greece, 2021-2023 based on top models' variables (microhabitat, local 250 m., and landscape 1km). Models included top detection model (month); represented as *p*(top), and top initial occupancy model (SH+R+LRiv1000); represented as Ψ(top). Extinction (ε) was held constant in all models. Models are ranked based on the lowest Akaike's Information Criterion (*AIC*) where *deltaAIC* = AICi – minimum AIC, *nPars* = number of parameters, and *AICwt* = AIC weight.

Model	AIC	deltaAIC	nPars	AICwt
**Ψ(top) γ(SH+RE) ε(.) *p*(top)**	**729,46**	**0,00**	**15**	**0,43**
Ψ(top) γ(SH+Arable250) ε(.) *p*(top)	729,72	0,26	15	0,37
Ψ(top) γ(SH+Shrub250) ε(.) *p*(top)	732,23	2,77	15	0,11
Ψ(top) γ(SH) ε(.) *p*(top)	735,18	5,72	14	0,03
Ψ(top) γ(SH+Dfarm) ε(.) *p*(top)	736,62	7,16	15	0,01
Ψ(top) γ(Dfarm+LRiv1000) ε(.) *p*(top)	736,65	7,19	15	0,01
Ψ(top) γ(SH+LRiv1000) ε(.) *p*(top)	737,17	7,71	15	0,01
Ψ(top) γ(RE+Dfarm) ε(.) *p*(top)	737,50	8,04	15	0,01
Ψ(top) γ(Shrub250+Arable250) ε(.) *p*(top)	738,07	8,61	15	0,00
Ψ(top) γ(RE) ε(.) *p*(top)	738,88	9,42	14	0,01
Ψ(top) γ(Shrub250) ε(.) *p*(top)	739,70	10,24	14	0,00
Ψ(top) γ(RE+Arable250) ε(.) *p*(top)	739,84	10,38	15	0,00
Ψ(top) γ(Arable250) ε(.) *p*(top)	740,09	10,63	14	0,00
Ψ(top) γ(null) ε(.) *p*(top)	740,20	10,74	13	0,01
Ψ(top) γ(RE+LRiv1000) ε(.) *p*(top)	740,88	11,42	15	0,00
Ψ(top) γ(Dfarm) ε(.) *p*(top)	741,01	11,55	14	0,00
Ψ(top) γ(RE+Shrub250) ε(.) *p*(top)	741,28	11,82	15	0,00
Ψ(top) γ(LRiv1000) ε(.) *p*(top)	742,11	12,65	14	0,00

Supplementary Table S11. Model results for probability of extinction (ε) from multi-season occupancy models of American mink in western Macedonia, Greece, 2021-2023 based on yearly covariates. Models included top detection model (month); represented as *p*(top), top initial occupancy model (SH+R+LRiv1000); represented as Ψ(top), and top colonization model (SH+RE); represented as γ(top). Models are ranked based on the lowest Akaike's Information Criterion (*AIC*) where *deltaAIC* = AICi – minimum AIC, *nPars* = number of parameters, and *AICwt* = AIC weight.

Model	AIC	deltaAIC	nPars	AICwt
**Ψ(top) γ(top) ε(catch) *p*(top)**	**729,36**	**0,00**	**16**	**0,33**
Ψ(top) γ(top) ε(null) *p*(top)	729,46	0,09	15	0,31
Ψ(top) γ(top) ε(S) *p*(top)	730,05	0,68	17	0,24
Ψ(top) γ(top) ε(trap) *p*(top)	731,36	1,99	16	0,12

Supplementary Table S12. Model results for probability of extinction (ε) from multi-season occupancy models of American mink in western Macedonia, Greece, 2021-2023 based on microhabitat variables. Models included top detection model (month); represented as *p*(top), top initial occupancy model (SH+R+LRiv1000); represented as Ψ(top), and top colonization model (SH+RE); represented as γ(top). Models are ranked based on the lowest Akaike's Information Criterion (*AIC*) where *deltaAIC* = AICi – minimum AIC, *nPars* = number of parameters, and *AICwt* = AIC weight.

Model	AIC	deltaAIC	nPars	AICwt
**Ψ(top) γ(top) ε(DEM) *p*(top)**	**727,07**	**0,00**	**16**	**0,26**
Ψ(top) γ(top) ε(DEM+RE) *p*(top)	727,68	0,61	17	0,20
Ψ(top) γ(top) ε(DEM+Strata3) *p*(top)	728,18	1,11	17	0,15
Ψ(top) γ(top) ε(null) *p*(top)	729,46	2,39	15	0,08
Ψ(top) γ(top) ε(RE) *p*(top)	729,62	2,55	16	0,07
Ψ(top) γ(top) ε(Strata3) *p*(top)	730,33	3,25	16	0,05
Ψ(top) γ(top) ε(GS) *p*(top)	730,75	3,68	16	0,05
Ψ(top) γ(top) ε(RE+Strata3) *p*(top)	731,12	4,05	17	0,03
Ψ(top) γ(top) ε(R) *p*(top)	731,43	4,35	16	0,03
Ψ(top) γ(top) ε(Strata2) *p*(top)	731,44	4,37	16	0,03
Ψ(top) γ(top) ε(BS) *p*(top)	731,46	4,39	16	0,03
Ψ(top) γ(top) ε(SH) *p*(top)	732,25	5,18	16	0,02

Supplementary Table S13. Model results for probability of extinction (ε) from multi-season occupancy models of American mink in western Macedonia, Greece, 2021-2023 based on local 250m variables. Models included top detection model (month); represented as *p*(top), top initial occupancy model (SH+R+LRiv1000); represented as Ψ(top), and top colonization model (SH+RE); represented as γ(top). Models are ranked based on the lowest Akaike's Information Criterion (*AIC*) where *deltaAIC* = AICi – minimum AIC, *nPars* = number of parameters, and *AICwt* = AIC weight.

Model	AIC	deltaAIC	nPars	AICwt
**Ψ(top) γ(top) ε(null) *p*(top)**	**729,46**	**0,00**	**15**	**0,18**
Ψ(top) γ(top) ε(Arable250) *p*(top)	729,89	0,43	16	0,15
Ψ(top) γ(top) ε(Dev250) *p*(top)	730,40	0,94	16	0,11
Ψ(top) γ(top) ε(Riv250) *p*(top)	730,55	1,09	16	0,10
Ψ(top) γ(top) ε(Shrub250) *p*(top)	730,64	1,18	16	0,10
Ψ(top) γ(top) ε(Arable250+Dev250) *p*(top)	731,05	1,59	17	0,09
Ψ(top) γ(top) ε(Dev250+Riv250) *p*(top)	731,07	1,61	17	0,08
Ψ(top) γ(top) ε(Broad250) *p*(top)	731,35	1,89	16	0,07
Ψ(top) γ(top) ε(HetAgri250) *p*(top)	731,44	1,98	16	0,06
Ψ(top) γ(top) ε(Arable250+Riv250) *p*(top)	731,76	2,30	17	0,06

Supplementary Table S14. Model results for probability of extinction (ε) from multi-season occupancy models of American mink in western Macedonia, Greece, 2021-2023 based on landscape 1km variables. Models included top detection model (month); represented as *p*(top), top initial occupancy model (SH+R+LRiv1000); represented as Ψ(top), and top colonization model (SH+RE); represented as γ(top). Models are ranked based on the lowest Akaike's Information Criterion (*AIC*) where *deltaAIC* = AICi – minimum AIC, *nPars* = number of parameters, and *AICwt* = AIC weight.

Model	AIC	deltaAIC	nPars	AICwt
**Ψ(top) γ(top) ε(Dfarm) *p*(top)**	**723,40**	**0,00**	**16**	**0,55**
Ψ(top) γ(top) ε(Dfarm+LRiv1000) *p*(top)	725,33	1,93	17	0,21
Ψ(top) γ(top) ε(Arable1000) *p*(top)	727,69	4,29	16	0,07
Ψ(top) γ(top) ε(LRiv1000) *p*(top)	728,63	5,23	16	0,04
Ψ(top) γ(top) ε(Arable1000+LRiv1000) *p*(top)	728,87	5,47	17	0,04
Ψ(top) γ(top) ε(null) *p*(top)	729,46	6,06	15	0,02
Ψ(top) γ(top) ε(Dfarm+Arable1000) *p*(top)	729,93	6,53	17	0,02
Ψ(top) γ(top) ε(Broad1000) *p*(top)	730,94	7,54	16	0,02
Ψ(top) γ(top) ε(Dev1000) *p*(top)	731,07	7,67	16	0,01
Ψ(top) γ(top) ε(Shrub1000) *p*(top)	731,34	7,94	16	0,01
Ψ(top) γ(top) ε(SRiv1000) *p*(top)	731,38	7,98	16	0,01
Ψ(top) γ(top) ε(HetAgri1000) *p*(top)	735,77	12,37	16	0,00

Supplementary Table S15. Model results for probability of extinction (ε) from multi-season occupancy models of American mink in western Macedonia, Greece, 2021-2023 based on top models' variables (microhabitat, local 250 m., landscape 1km, and yearly). Models included top detection model (month); represented as *p*(top), top initial occupancy model (SH+R+LRiv1000); represented as Ψ(top), and top colonization model (SH+RE); represented as γ(top). Models are ranked based on the lowest Akaike's Information Criterion (*AIC*) where *deltaAIC* = AICi – minimum AIC, *nPars* = number of parameters, and *AICwt* = AIC weight.

Model	AIC	deltaAIC	nPars	AICwt
**Ψ(top) γ(top) ε(Dfarm) *p*(top)**	**723,40**	**0,00**	**16**	**0,63**
Ψ(top) γ(top) ε(catch+DEM) *p*(top)	726,71	3,31	17	0,12
Ψ(top) γ(top) ε(DEM) *p*(top)	727,07	3,67	16	0,11
Ψ(top) γ(top) ε(DEM+Dfarm) *p*(top)	728,58	5,17	17	0,04
Ψ(top) γ(top) ε(catch+Dfarm) *p*(top)	729,27	5,87	17	0,04
Ψ(top) γ(top) ε(catch) *p*(top)	729,36	5,96	16	0,03
Ψ(top) γ(top) ε(null) *p*(top)	729,46	6,06	15	0,03
